# Missed opportunities for ovarian salvage in children: an 8-year review of surgically managed ovarian lesions at a tertiary pediatric surgery centre 

**DOI:** 10.1007/s00383-021-04935-w

**Published:** 2021-07-07

**Authors:** D Karavadara, JR Davidson, L Story, Y Diab, M Upadhyaya

**Affiliations:** 1 Department of Paediatric Surgery, Evelina London Children’s Hospital, Lambeth Palace Road, London, SE1 7EH UK; 2 King’s College London School of Medicine, London, UK; 3 Stem Cells and Regenerative Medicine, GOS-UCL Institute of Child Health, London, UK; 4 Prenatal Cell and Gene Therapy, EGA-UCL Institute for Women’s Health, London, UK; 5 Department of Obstetrics, Gynaecology and Maternal-Fetal Medicine, Guy’s and St Thomas’ NHS Foundation Trust, London, UK

**Keywords:** Ovarian sparing surgery, Pediatric ovarian lesions, Antenatal diagnosis, Ovarian loss, OSS

## Abstract

**Introduction:**

The aetiology and management of ovarian pathology in children differs between antenatal and postnatal lesions. However, all lesions may present acutely due to adnexal torsion. In this setting, opportunities to preserve fertility with ovary-sparing surgery (OSS) may be missed. Some studies suggest that pediatric and adolescent gynaecology (PAG) input in care is associated with OSS.

**Methods:**

A retrospective cohort study of children undergoing surgery for ovarian pathology at a tertiary pediatric surgery centre over an 8-year period (2011–2018). Patient factors, lesion characteristics and PAG involvement were examined for association with OSS using multivariate logistic regression.

**Results:**

Thirty-five patients with ovarian pathology managed surgically were included. Ten were infants with lesions detected antenatally; all were managed by pediatric surgeons (PS) alone at median age 2 weeks (1 day–25 weeks). Twenty-five patients presented postnatally at median age 11 (0.75–15) years. In total, there were 16 cases of adnexal torsion, each managed primarily by PS. Twelve underwent oophorectomy and six (50%) of these cases had viable ovarian tissue on histology. Furthermore, two infants with large simple cysts were similarly managed by unnecessary oophorectomy based on histology. Overall rate of OSS was 46% and PAG involvement was the only factor associated with ovarian salvage.

**Conclusion:**

Differences in surgical management between PAGs and PS may be attributable to the different patient populations they serve. We recommend improving the knowledge of PS trainees in OSS approaches for adnexal torsion and large benign lesions.

## Introduction

Ovarian lesions in children are uncommon. However, children of all ages may present with ovarian pathology. In female neonates, ovarian cysts are the most common cause of cystic abdominal masses seen on ultrasound scan (USS) [1]. These are usually simple cysts that develop in utero in response to follicular stimulation by maternal and placental hormones in the fetal circulation. They, therefore, usually regress postnatally. In the older child, benign and malignant ovarian neoplasms are more prevalent [2, 3]. Paraovarian cysts, anatomically distinct from the ovaries, have an incidence of 7.3% in children [4]. All ovarian lesions, including paraovarian cysts, can induce adnexal torsion and are, therefore, linked with a risk of ovarian loss secondary to ischaemia or surgical resection [5].

Surgical intervention is indicated if there is a high suspicion for malignancy, if benign lesions are large and persistent or if lesions are complicated by adnexal torsion [6]. In addition, current evidence based on retrospective data suggests that neonatal ovarian cysts larger than 40 mm are significantly less likely to spontaneously resolve and more likely to induce adnexal torsion [7, 8]. In these cases, surgery may be performed pre-emptively.

Surgical management of ovarian lesions is by oophorectomy or by ovary-sparing surgery (OSS), to preserve viable ovarian tissue and fertility. OSS may include combinations of simple detorsion, needle aspiration, cyst fenestration (with/without marsupialisation), cystectomy and oopexy. Removal of a single ovary is linked with a 32% decrease in the odds of successful assisted reproduction [OR = 0.68 (0.51–0.92), *p* = 0.012] [9], more than a threefold increase in the risk of premature ovarian failure [OR = 3.32 (1.42–7.77)] and early menopause [OR = 3.94 (2.63–5.89)] [10], as well as an increased risk of surgical castration following contralateral adnexal torsion or ovarian neoplasia [11, 12]. Therefore, ovarian lesions with features suggestive of benign pathology should be managed by OSS wherever possible.

Care for children with ovarian lesions is usually provided by pediatric surgeons (PS) and/or pediatric and adolescent gynaecologists (PAG). Some studies have indicated differences between these two specialties in their surgical approach to ovarian lesions in children, where PAG involvement is more likely to result in OSS [13–17].

This retrospective cohort study examined patients aged under 18 years with ovarian pathology and undergoing surgery at a tertiary pediatric surgery centre over an 8-year period. We hoped to determine our rate of OSS and to identify factors associated with ovarian salvage as well as missed opportunities for fertility preservation.

## Methods

### Study design

After institutional ethical approval (Ref: 7982), a retrospective chart review was performed for all patients aged under 18 years presenting to our unit with ovarian pathology between January 2011 and December 2018. Evelina London Children’s Hospital is a regional referral centre for pediatric care, except it does not treat children with suspected or confirmed oncologic diagnoses. It also shares a site with St. Thomas’ Hospital—a regional referral centre for fetal medicine, obstetrics and gynaecology and gynae-oncology.

Patients were identified using a prospectively maintained departmental logbook, which was verified against our institutional fetal medicine database and records from our pathology department. Data were collected on demographics, clinical presentation, pre-operative imaging, intra-operative details, including specialist involvement and procedure performed, as well as histological findings.

### Definitions

With respect to imaging, a complex cyst was differentiated from a simple cyst using the following features reported by the observer: the presence of solid components, heterogeneous echogenicity, internal septations, mural papillary projections and/or increased mural vascularity.

### Outcomes

Our primary outcome was to determine the rate of OSS and to identify factors affecting this using a multivariate model. A range of variables were examined, including patient age, acuity of presentation, appearances of the lesion on pre-operative imaging and intra-operatively, as well as the contribution of each specialist to patient care. We also set out to identify any missed opportunities for ovarian salvage by looking for any histological evidence of viability in cases managed by oophorectomy.

### Analysis

Data are presented as median with interquartile range (IQR) throughout the document unless otherwise stated. Statistical analyses were performed on SPSS 26.0 (IBM™) using Fisher’s exact test and binomial logistic regression for outcomes of OSS; categorisation of continuous variables was performed based on observed distribution of raw data and these were defined prior to performing any analysis of outcome. Statistical significance was determined as a *p* value < 0.05.

## Results

### Antenatally detected lesions

Twenty-nine patients were referred to our antenatal counselling clinic for adnexal anomalies detected on routine USS. Five patients were not followed up: one pregnancy miscarried, and four patients were born at locations outside our surgical network. In three patients, repeat USS in the third trimester showed resolution of the lesion in utero. Another five patients had non-ovarian pathology on postnatal USS, including perforated ileal atresia and an ileal duplication cyst. This left 16 patients who had true ovarian pathology persisting postnatally: six were managed conservatively with serial USS and ten underwent surgery at a median age of 2 weeks (range 1 day–25 weeks, Table 1).

**Table 1:** Surgical management and histological diagnosis of antenatally detected lesions

	Total patients (*N* = 10), *n *(%)
Indications for surgery	
Emergency presentation	2 (20%)
Lesion size or appearance	7 (70%)
Size > 40 mm	6
Complex	3
Unknown	1 (10%)
Specialist involvement	
Pediatric surgeon	10 (100%)
Intra-operative findings	
Lesion appearance	
Simple	10 (100%)
Adnexal torsion	5 (50%)
Surgical procedure	
Oophorectomy	6 (60%)
OSS (cystectomy)	4 (40%)
Surgical technique	
Laparoscopic	2 (20%)
Open conversion	1 (10%)
Open	7 (70%)
Histological diagnosis (*N* = 10)	
Follicular cyst	4 (40%)
Corpus luteal cyst	2 (20%)
Indeterminate (due to torsion)	4 (40%)

Two patients (20%) presented acutely: one presented generally unwell and the other with a palpable abdominal mass and vomiting. On pre-operative imaging, eight lesions (80%) were larger than 40 mm [median lesion size = 76 mm (55–93 mm)] and three lesions (30%) had a complex appearance.

All ten antenatally detected lesions were managed by PS without any PAG involvement. Four lesions (40%) were managed by ovarian cystectomy and six (60%) by oophorectomy. Of those undergoing oophorectomy, four were complicated by adnexal torsion and the other two were large simple cysts (98 and 100 mm on pre-operative imaging) with no identifiable normal ovarian tissue intra-operatively. Histological examination showed that these two cysts and two cases of adnexal torsion may have been managed by unnecessary oophorectomy given the presence of viable ovarian follicles.

### Postnatal presentations

Twenty-five patients presenting postnatally underwent surgery at a median age of 11 (range 0.75–15) years (Table 2). Sixteen patients (64%) had emergency surgery having presented acutely with symptoms: 14 (88%) presented with abdominal pain, nine (56%) with vomiting and five (31%) with a palpable abdominal mass. Two patients presented with cognitive disturbance; both were ultimately diagnosed with mature cystic teratomas, so this was likely secondary to autoimmune encephalitis.

**Table 2:** Surgical management and histological diagnosis of patients presenting postnatally

	Total patients (*N* = 25), *n *(%)
Presentation	
Emergent	16 (64)
Elective	9 (36)
Specialist involvement	
Pediatric surgeon	17 (68)
Pediatric surgeon with PAG assistance	5 (20)
PAG only	3 (12)
Intra-operative findings	
Lesion appearance	
Simple	10 (40)
Complex	13 (52)
Adnexal torsion	11 (44)
Surgical procedure	
Oophorectomy	13 (52)
OSS	12 (48)
Aspiration only	2 (8)
Fenestration	4 (16)
Cystectomy	5 (2)
Oopexy	1 (4)
Surgical technique	
Laparoscopic	14 (56)
Open conversion	4 (16)
Open	7 (28)
Histological diagnosis (*N* = 22)	
Follicular cyst	1 (5)
Corpus luteal cyst	3 (13)
Mature cystic teratoma	13 (59)
Immature cystic teratoma	1 (5)
Massive ovarian oedema	1 (5)
Indeterminate (due to torsion)	3 (13)

All but one patient (24/25, 96%) had pre-operative imaging; three (12%) had CT, fourteen (56%) had MRI and eight had an USS alone. Median lesion size was 75 mm (45–147 mm). Nineteen lesions (76%) appeared complex on pre-operative imaging. The only patient without imaging underwent a diagnostic laparoscopy for suspected appendicitis, where intra-operative findings were of a right simple ovarian cyst without adnexal torsion.

All 16 patients undergoing emergency surgery were managed primarily by PS. PAG assistance in theatre was received for three of these cases (19%) and PAG advice was sought in one other elective case. The three cases managed independently by PAGs were elective procedures.

Twelve cases (48%) were managed by OSS and 13 (52%) by oophorectomy. Of those undergoing oophorectomy, eight were complicated by adnexal torsion and the other five were complex ovarian lesions that were large (18 and 20 cm on pre-operative imaging) or with no discernible normal ovarian tissue intra-operatively. Histological examination showed that four cases of adnexal torsion may have been managed by unnecessary oophorectomy given the presence of viable ovarian follicles or the absence of tissue ischaemia/infarction.

Specimens were sent for histological examination for 22 ovarian lesions—three were managed without tissue resection (oopexy or needle aspiration only). Mature cystic teratomas were the most common histological finding, diagnosed in 13 lesions. There was only one lesion with malignant potential, which was an immature cystic teratoma. Not shown in Table 2 is that in three patients a concurrent paraovarian lesion was found in addition to a true ovarian lesion: one simple cyst and the two benign serous cystadenomas, which were managed by fenestration and cystectomy, respectively.

The only known lesion recurrence occurred in a 15-year-old girl presenting electively with a left simple ovarian cyst and a contralateral paraovarian serous cystadenoma. These were initially managed by laparoscopic aspiration and cystectomy, respectively. She required a repeat open cystectomy plus lymph node sampling and omentectomy 11 months later for the right paraovarian lesion. Histology showed a benign serous cystadenofibroma without evidence of malignancy.

### Multivariate analysis

All 35 patients were included in the multivariate analysis to assess for factors associated with OSS (Table 3). PAG involvement in surgical care was the only factor significantly and independently associated with OSS [adjusted OR = 93.9 (2.5–3503), *p* = 0.014].

**Table 3:** Factors associated with OSS

Factor	Adjusted OR (95% CI)	*p* value
Age > 1 year	0.18 (0.01–1.06)	0.250
Elective procedure	2.79 (0.20–38.4)	0.442
**PAG involvement**	**93.9 (2.5–3503)**	**0.014**
Complex appearance	0.04 (0.001–1.064)	0.054
Adnexal torsion	0.15 (0.12–1.80)	0.133

Statistical significance indicated in bold

*OR* odds ratio, *CI* confidence interval

## Discussion

This study reviewed the management of 35 patients presenting with ovarian pathology at a pediatric surgery centre over an 8-year period. The rate of OSS was 46% (16/35), which is within the range of ovarian conservation rates reported elsewhere [18]. In keeping with a recent literature review [19], we show that PAG involvement is associated with OSS. However, we also report significant differences in the patient populations served by PAGs compared with PS. Most notably, all cases of adnexal torsion were managed by PS, with PAGs providing surgical assistance in one instance. Furthermore, we found that, given one exception, PAGs were only involved in the care of patients over 10 years old. Several other studies have similarly shown that older patients are significantly more likely to receive care from PAGs [13, 16–18]. It has been speculated that post-menarche, patients are more likely to have accessed gynaecology services before and are, therefore, more inclined to present or to be referred to PAGs with ovarian pathology. Overall, this makes it difficult to compare the surgical practices of these specialties against each other.

There were 16 cases of adnexal torsion in this study. Twelve cases (75%) were managed by oophorectomy; only five of these cases had a definitive histological confirmation of pathology, four of which were cystic teratomas (one immature). In six cases (50%) there was evidence of viable ovarian tissue on histology, indicating that these may have been missed opportunities for ovarian salvage. Historically, adnexal torsion has been managed by oophorectomy based on the belief that tissue necrosis seen intra-operatively indicates poor ovarian viability. This has repeatedly been shown to be untrue. In a retrospective review of 29 girls presenting with adnexal torsion and undergoing detorsion, 97% had ovarian follicles seen on the affected side on follow-up USS [20]. As a result, primary detorsion is now widely considered first-line management of adnexal torsion. After detorsion, any benign lesions of either adnexa may also be removed during the same procedure to reduce the risk of contralateral recurrence [21]. For example, here, three cases of adnexal torsion in this study were managed by primary detorsion followed by fenestration of a follicular cyst in one case and resection of a benign complex lesion in the other two cases (one was a benign serous cystadenoma confirmed on histology).

One case of adnexal torsion in this study was managed by oopexy after detorsion and aspiration of a simple ovarian cyst. Evidence shows that there is an ongoing risk of recurrent adnexal torsion even after adnexal fixation [22]. Also, in the presence of a benign adnexal lesion, there is no significant difference in recurrence risk after oopexy compared to detorsion plus fenestration or resection of the lesion [21]. There may, however, be a role for oopexy in the management of adnexal torsion with long utero-ovarian ligaments, in cases of recurrence or where there is a risk of castration, such as with bilateral adnexal torsion or previous unilateral oophorectomy.

Two infants with large, antenatally detected simple cysts were managed by oophorectomy in this cohort. In each case, there was no identifiable normal ovarian tissue intra-operatively. Unfortunately, histological examination showed that these were also missed opportunities for ovarian salvage. OSS in cases where ovarian tissue cannot be distinguished macroscopically may be achieved utilising the stripping technique, which has been shown to be effective in children with benign ovarian lesions [23].

The only potentially malignant lesion in our cohort was a torted immature cystic teratoma in a 9-month-old girl. This was managed by open oophorectomy (post-operative care was transferred to a pediatric oncology centre). Even in the presence of a suspicious ovarian mass, some groups advocate initial primary detorsion with tissue biopsy for confirmation of diagnosis [24]. Definitive surgery could then be performed in an elective setting following risk stratification of the mass [2].

Limitations of this study include those related to its single centre, retrospective design, with inherent selection bias by the fact that our centre sees fetal medicine referrals from the region and does not manage pediatric cancer. For example, we report only one lesion with malignant potential (3%), which contrasts with an expected malignancy rate of approximately 10% [25]. Our data was also collected from a relatively small cohort of patients, which is not unsurprising given the frequency of presentation with ovarian pathology in the pediatric age range. This study, therefore, lacks statistical power, compared to those using national-level data [13], and may be prone to a Type II statistical error. On the other hand, patients with antenatally detected lesions are better represented here than elsewhere and we believe the granularity of our collected data has allowed for a meaningful analysis of variables associated with OSS. Importantly it should be recognised that there are clear differences in patient’s characteristics between PS and PAGs. As such, the fact that PAGs is a significant factor on a multivariate analysis with a cohort this size would likely represent that it is simply the strongest statistical factor analysed rather than the only significant factor, and as such needs to be interpreted with caution prior to validation in a larger dataset.

In conclusion, this study shows that although differences in surgical management exist between PAGs and PS, these may be attributable to the different patient populations they serve. In addition, while PAG input in cases led by PS may be helpful, this is often not feasible in the acute setting. Therefore, to improve fertility preservation in children presenting with ovarian pathology, we recommend improving the knowledge of PS trainees in OSS approaches for adnexal torsion and large benign lesions.
